# Use of an electronic medical record reminder improves HIV screening

**DOI:** 10.1186/s12913-017-2824-9

**Published:** 2018-01-10

**Authors:** Colleen Kershaw, Jessica L. Taylor, Gary Horowitz, Diane Brockmeyer, Howard Libman, Gila Kriegel, Long Ngo

**Affiliations:** 1 Department of Medicine, Beth Israel Deaconess Medical Center, 110 Francis Street, Suite GB, Boston, MA 02215 USA; 2 Department of Medicine, Boston University School of Medicine, 801 Massachusetts Avenue, Crosstown 2, Boston, MA 02118 USA; 3 Department of Pathology, Tufts Medical Center, Biewend Building 3, 800 Washington St, Boston, MA 02111 USA; 4 Department of Medicine, Beth Israel Deaconess Medical Center, 330 Brookline Avenue, Boston, MA 02215 USA

**Keywords:** HIV, Electronic medical record reminder, Decision support, Screening

## Abstract

**Background:**

More than 1 in 7 patients with human immunodeficiency virus (HIV) infection in the United States are unaware of their serostatus despite recommendations of US agencies that all adults through age 65 be screened for HIV at least once. To facilitate universal screening, an electronic medical record (EMR) reminder was created for our primary care practice. Screening rates before and after implementation were assessed to determine the impact of the reminder on screening rates.

**Methods:**

A retrospective cohort analysis was performed for patients age 18–65 with visits between January 1, 2012-October 30, 2014. EMR databases were examined for HIV testing and selected patient characteristics. We evaluated the probability of HIV screening in unscreened patients before and after the reminder and used a multivariable generalized linear model to test the association between likelihood of HIV testing and specific patient characteristics.

**Results:**

Prior to the reminder, the probability of receiving an HIV test for previously unscreened patients was 15.3%. This increased to 30.7% after the reminder (RR 2.02, CI 1.95–2.09, *p* < 0.0001). The impact was most significant in patients age 45–65. White race, English as primary language, and higher median household income were associated with lower likelihoods of screening both before and after implementation (RR 0.68, CI 0.65–0.72; RR 0.74, CI 0.67–0.82; RR 0.84, CI 0.80–0.88, respectively).

**Conclusions:**

The EMR reminder increased rates of HIV screening twofold in our practice. It was most effective in increasing screening rates in older patients. Patients who were white, English-speaking, and had higher incomes were less likely to be screened for HIV both before and after the reminder.

## Background

There are estimated to be 1,200,000 HIV-infected people in the United States [1]. Advances in treatment have rendered HIV a manageable chronic illness in most patients; however, an estimated 14% of HIV-infected persons in the US remain undiagnosed [1]. The Centers for Disease Control and Prevention (CDC) and the United States Preventive Services Task Force (USPSTF) recommend that all adolescents and adults up to age 65 be screened at least once for HIV regardless of risk behaviors [2, 3]. However, significant challenges remain in implementing published guidelines [4].

Health systems and individual practitioners are increasingly evaluated on their adherence to screening recommendations, and reimbursement is predicated on meaningful use of newer technologies such as the electronic medical record (EMR). EMR reminders have become a common method for encouraging practitioners to meet screening guidelines. Although some practitioners have raised concern about “alert fatigue,” the literature suggests that EMR reminders improve screening rates for many conditions, including breast cancer, osteoporosis, abdominal aortic aneurysms, and obesity [5–8]. A recent study on hepatitis C screening has also shown an electronic reminder to be successful [9]. However, data on HIV screening remain mixed, with several studies reporting increases in screening with EMR reminders in combination with other interventions [10–12], while at least one study did not show an increase with electronic reminders [13].

In October 2013, Beth Israel Deaconess Medical Center, a large academic medical center in Boston, MA, enacted a passive EMR reminder for HIV screening in all patients age 13–65. The reminder, which reads, “HIV antibody,” appears on the home page of each patient’s EMR in small red text alongside other clinical reminders. If a test is performed, the reminder disappears automatically. If a test has been done at an outside institution or if the patient declines testing, the practitioner can manually enter this information, causing the reminder to disappear; however, this manual entry is not consistently performed.

The goal of this study was to evaluate the impact of the EMR reminder on rates of screening for HIV at an academic, hospital-based primary care practice. A secondary aim was to identify patient characteristics associated with the likelihood of HIV screening.

## Methods

### Design

A retrospective cohort analysis was performed for all patients seen at the practice between January 1, 2012 and October 30, 2015. The study was approved by the Beth Israel Deaconess Medical Center Institutional Review Board on August 15, 2014 (protocol #2014P000210).

Patients were included if they fell between the ages of 18–65 (in our adult internal medicine practice) and had at least one visit in the practice during the 2.5-year study period. They were excluded if they did not have demographic information available. Age, gender, socioeconomic status (determined by zip code), primary language, and number of visits in the study period were abstracted. Patient records were assessed for presence or absence of an HIV antibody test before or after implementation of the EMR screening prompt on October 30, 2013.

Billing and laboratory databases were also used to abstract the number of HIV tests that practitioners ordered per month during the study period. Proportions of HIV testing before and after the EMR prompt implementation were compared.

Patients were included in the “before” group if they had a visit during the period of January 1, 2012 through October 29, 2013 and had not previously been tested for HIV. Patients were included in the “after” group if they had a visit in the practice between October 30, 2013 and October 30, 2015 and had not previously been tested for HIV (considered “at risk”).

During the study period, no other changes were made to the “Reminders” section of our electronic medical record, nor was there any specific initiative to highlight reminders as part of Meaningful Use trainings.

### Statistical analysis

There were two main analyses performed for this study. The first was to compare the proportion of previously unscreened patients receiving an HIV test prior to October 30, 2013 (the “before” group) to the proportion receiving an HIV test after October 30, 2013 (the “after” group). A patient could appear in both proportions if he or she had at least one visit in the “before” period, did not have HIV testing in the “before” period, and had a visit in the “after” period. Because of this possibility, the observations used in the analysis were not independent in the comparison of the two sample proportions (“before” vs “after”). We took into account the within-subject correlation in the data by using a generalized estimating equations (GEE) with log link and binary error. We used the log link, which yields relative risks, instead of logit link, which yields odds ratios, because the probability of HIV testing exceeded 10% in the sample and thus odds ratios were not a good approximation of the relative risks. We used exchangeable working correlation structure in the GEE model and linear contrasts to obtain the relative risk estimate and its 95% confidence interval.

In the second main analysis, we modeled the associations between HIV testing (binary outcome) and patient demographics (age, gender, race, language, income) using a GEE model similar to the first analysis. For the multivariate analysis of the effect of these patient characteristics on likelihood of overall testing, given our very large “N,” *p*-values for all factors were expected to be statistically significant. We were not interested in prediction, but rather the explanation of the association of each of the available patient characteristics to HIV testing, and thus we used all the available factors in the generalized estimating equations (GEE) with log link for relative risks estimates.

We then performed additional analyses looking at effects of health services utilization on HIV screening. We examined whether the duration of time enrolled in the practice for each group (“before” and “after”) may have affected the outcome by comparing the median time of enrollment between each group. We computed the time enrolled in care within the practice by pulling all visit dates from each patient’s first visit in the practice until November 1, 2014. The time of enrollment was the difference between the first and last visit date. We also examined the frequency of patient visits and compared this between the study periods.

Two sensitivity analyses were performed to assess the impact of different modeling approaches on the intervention effect. First, we performed a time-to-event survival analysis. For all patients in both the “before” and “after” time periods, we computed survival time as the duration from the first-ever visit to the date when an HIV test was done. This was used as the “survival time” for non-censored subjects. For those patients who did not have HIV testing (censored observations), the survival time was the difference between first visit and the end of follow up time. For the “before” group, the date of activation of the screening reminder (October 30, 2013) served as the censorship date. For the “after” group, the date of censorship was October 30, 2014 (end of follow-up time.) We ran Cox’s proportional hazards models comparing the rate of HIV testing (hazard) between the “before” and “after” period, with the “before” period serving as a reference in the model. The test of median difference was used to determine whether there was a significant difference.

The second sensitivity analysis examined only *new* patients within the practice, with no overlap of patients between the two time periods, in order to evaluate for the possibility of underlying, unmeasured characteristics of patients who were not screened in the “before” period, which also may have made them unlikely to be screened in the “after” period.

Because we hypothesized that providers with different levels of experience may adopt new screening recommendations at different rates, we did stratification analysis by provider types (attending physicians versus resident physicians) in order to obtain the absolute risk estimates for each of four possible combinations of the interaction between time period and provider type. We reported these as percent estimates. We then looked at the modification effect of the intervention by provider type, by performing modeling with an interaction term between time period (1: pre, 2: post) and provider type. We used GEE model with log link and binomial error term. We also used linear contrasts of the model parameters to estimate the relative risks and their corresponding 95% confidence intervals.

Finally, Massachusetts law changed on June 26, 2012 and voided a previous requirement for written consent for HIV testing. The added requirement of obtaining written consent during the first 6 months of our “before” period may have confounded our analyses, so we performed an additional analysis which removed the first 6 months of the “before” period.

Analyses used data with non-missing demographics; all data including missing and non-missing demographics accounted for *N* = 63,725 (35,945 in the before period, and 27,780 in the after period). Non-missing demographics yielded an *N* = 48,369 (27,729 in the before, and 20,640 in the after). We used the SAS/STAT software version 9.3 for all data management and statistical analyses of this study.

## Results

### Patient characteristics

In total, 27,729 patients in the “before” period and 20,640 in the “after” period met inclusion criteria. These two periods were not mutually exclusive. Patients who had visits both before and after reminder implementation (but had not been tested for HIV at the beginning of either study period) were included in both groups. The patients accounted for 63,725 visits within the 2.5-year study period. The majority were age 46–65, female, and spoke English as their primary language (Table 1).

**Table 1:** Patient demographics

	Patients with Visits Before (27,729) N (%)	Patients with Visits After (20,640) N (%)
Age < 25	2289 (8.3)	1715 (8.3)
Age 26–35	6924 (25)	4508 (21.8)
Age 36–45	5480 (19.8)	3904 (18.9)
Age 46–65	13,024 (47)	10,513 (50.9)
Female	16,314 (58.8)	12,040 (58.3)
Male	11,413 (41.2)	8600 (41.7)
Asian	1879 (6.8)	1526 (7.4)
Black	4644 (16.8)	3475 (16.8)
Other race	5073 (18.3)	2557 (12.4)
White	16,131 (58.2)	12,812 (62.1)
Primary language English	26,648 (96.1)	19,742 (95.7)
Primary language non-English	1079 (3.9)	898 (4.4)
Income (by zip code):		
Median	$67,338	$67,338
Mean	$70,831	$70,892
SD	$27,379	$27,328

### HIV testing prior to reminder

Before the reminder, 15.3% of patients who had not been previously screened received HIV testing. The practice performed an average of 254 HIV tests per month. Pre-reminder data showed a trend towards lower testing rates in older patients, with just 9.7% of 46–65 year-olds receiving testing compared to 15.9% of 36–45 year-olds, 23.1% of 26–35 year-olds, and 22.5% of patients <25 years old. Women were also less likely to be tested with 14.0% screened compared to 17.3% of men.

### HIV testing after reminder

After the reminder, 30.7% of patients who had never been tested for HIV received HIV testing, representing a 2.02 relative risk of screening as compared to the pre-reminder period (*p* < 0.0001, CI 1.95–2.09). The average number of HIV tests per month done in the practice increased from 254 to 462.

**Fig. 1:**
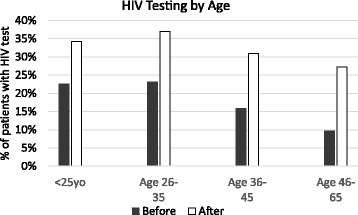
HIV Testing by Age. Differences in percentage of patients screened by age groups before and after the EMR prompt. Age < 25yo: 22.5 vs 34.3%, RR 1.52 (CI 1.38–1.69), *p* < 0.0001; Age 26–35: 23.1% vs 37%, RR 1.61 (CI 1.53–1.71), *p* < 0.0001; Age 36–45: 15.8% vs 31%, RR 1.98 (CI 1.83–2.13), *p* < 0.0001; Age 46–65: RR 2.81 (2.65–2.99), *p* < 0.0001

The increase in testing rates was observed in patients of all age, gender, race, language, and income groups (Table 2). One of the most striking effects showed that, although patients age 46–65 were least likely to be tested pre-reminder, the reminder had the largest effect in this older age group, increasing the proportion of patients tested from 9.7% to 27.2%, RR 2.81 (CI 2.65–2.99) (Fig. 1). Also notably, the reminder equalized rates of testing between genders after implementation, resulting in a 31% rate in both groups.

**Table 2:** HIV testing rates before and after EMR reminder implementation stratified by levels of patient characteristics

Patient characteristic	Proportion tested before reminder	Proportion tested after reminder	Relative Risk and 95% Confidence Interval^a^	*P*-value^a^
OVERALL	15.3%	30.7%	2.02 (1.95–2.09)	< 0.0001
AGE				
Age ≤ 25	22.5%	34.3%	1.52 (1.38–1.69)	<0.0001
Age 26–35	23.1%	36.9%	1.61 (1.53–1.71)	<0.0001
Age 36–45	15.8%	31.0%	1.98 (1.83–2.13)	<0.0001
Age 46–65	9.7%	27.2%	2.81 (2.65–2.99)	<0.0001
GENDER				
Female	14.0%	30.5%	2.19 (2.10–2.30)	<0.0001
Male	17.3%	30.8%	1.82 (1.73–1.91)	<0.0001
RACE				
White	13.9%	27.1%	1.97 (1.88–2.07)	<0.0001
Black	21.5%	41.0%	1.93 (1.81–2.06)	<0.0001
Asian	14.5%	30.3%	2.11 (1.85–2.40)	<0.0001
Other race	14.6%	33.4%	2.31 (2.12–2.51)	<0.0001
LANGUAGE				
English speaking	15.3%	30.4%	2.01 (1.94–2.08)	<0.0001
Non-English speaking	17.2%	37.5%	2.20 (1.89–2.56)	<0.0001
INCOME				
Income < median ($67,338)	19.1%	34.9%	2.28 (2.16–2.40)	<0.0001
Income > median ($67,338)	11.9%	26.9%	1.85 (1.77–1.93)	<0.0001

^a^Estimates from generalized estimating equations models where the dependent variable is HIV testing (binary 1 or 0), and the independent variable is time period (before, after reminder). The before reminder time period serves as the reference category, and stratification is used for each level of the patient characteristic

The increase in HIV testing rates also yielded more HIV diagnoses. In the period before the intervention, 0.3% of HIV tests were positive, as compared to 0.7% of tests after the reminder. This represents slightly more than a two-fold increase in HIV positivity rate in the after period (*p*-value = 0.0001).

### HIV testing overall

A multivariate regression was used to examine the effect of patient characteristics on the likelihood of HIV testing at the practice overall, incorporating both the pre-reminder and post-reminder time periods (Table 3). Patients age 25 or younger remained more likely to be tested for HIV compared to those in the 46–65 age group (used as the reference group for age), as were patients in the 26–35 age group (RR 1.65 with CI 1.51–1.80 and RR 1.71 with CI 1.62–1.80, respectively). Although testing rates for men and women seemed to equalize in the post-reminder period, men remained slightly more likely to have been tested in examining overall practice data, with a relative risk of 1.12 (*p* < 0.0001, CI 1.06–1.16). The reminder had a larger effect on increasing testing for females (RR 2.19) than males (RR1.82). Overall, white patients were least likely to be tested for HIV both before and after the reminder, which was particularly pronounced when comparing to the probability of HIV testing in black patients, with a relative risk of 0.68 (*p* < 0.0001, CI 0.65–0.72). English speaking patients were also less likely to be tested overall, with a relative risk of 0.74 (*p* < 0.001, CI 0.67–0.82). Patients who lived in zip codes with household incomes higher than the median of $67,338 were slightly less likely to be tested (RR 0.84, p < 0.0001, CI 0.80–0.88).

**Table 3:** Adjusted association between patient characteristics and HIV testing overall

Patient Characteristics	Relative Risk	Confidence Interval	*P* Value
Gender-male	1.12	1.06–1.16	*p* < 0.0001
White vs. Black race	0.68	0.65–0.72	*p* < 0.0001
White vs. Asian race	1.00	0.92–1.09	*p* = 0.9935
White vs. Other race	0.96	0.90–1.03	*p* = 0.2300
Age < 25 vs 45–65	1.65	1.51–1.80	*p* < 0.0001
Age 26–35 vs 45–65	1.71	1.62–1.80	*p* < 0.0001
Age 35–45 vs 46–65	1.29	1.22–1.37	*p* < 0.0001
English speaking vs other	0.74	0.67–0.82	*p* < 0.0001
Income >$67,338	0.84	0.80–0.88	*p* < 0.0001

Estimates from generalized estimating equations with log link. Reference group: Female, white, age:45–65, language: other, income: <=$65,338

### Effect of healthcare utilization on HIV testing

Median time enrolled in the practice for patients in the pre-reminder period was 2093 days, compared to 2286 days for patients in the post-reminder period. This was not statistically different between the study periods (*p* = 0.598). Survival analysis examining the duration of time in the practice from the first visit until the first HIV test showed that the hazard ratio for the rate of HIV testing between the “before” and “after” periods (with “before” as the reference period) was 1.92 (95% CI 1.84–1.99). This estimate suggests that in the post-reminder period, the rate of HIV testing was approximately twice that of the pre-reminder period. This is in agreement with our analysis using repeated measures modeling method of generalized estimating equations (GEE), where we obtained the relative risk of 2.02 and 95% confidence interval of 1.95–2.09 (Table 2).

In both time periods, patients enrolled in the practice for a shorter duration were more likely to receive an HIV test. In the “before” period, patients who had HIV testing were enrolled for a median time of 2.2 years, whereas those with no testing were enrolled for median time of 5.4 years. In the post- reminder period, patients who received an HIV test had been enrolled for a median of 4.5 years, as compared to 5.2 years among those who did not receive an HIV test.

There was no correlation between the number of visits the patient made to the practice and the likelihood of HIV testing, either before or after the reminder (Spearman coefficient = −0.03). The median number of visits was the same (10) in both the “before” (mean 15.48, SD 16.90) and “after” periods (mean 15.52, SD 17.57) (*p* = 0.065). Clinically, there is little difference between the two distributions of visit frequency.

### Sensitivity analysis: New patients

In the pre-reminder period, there were 7968 (out of total 27,729), or 29%, new patients, as compared to 7469 (36%) new patients in the post-reminder period. These two proportions are significantly different (*p* = 0.0001). Before the intervention, the HIV test proportion in new patients was 22.5% (1796/7968), compared to 32.7% (2441/7469) after the intervention, with relative risk of receiving an HIV test after the reminder of 1.46 (*p* = 0.0001). The rate of new patient screening was higher than the rate of screening for the overall study group in both the before (22.5% vs. 15.3%) and after (32.9% vs 30.7%) periods. However, there was a smaller change in the likelihood of HIV screening after the reminder among new patients.

### HIV testing by provider type

In the “before” period, our providers were comprised of 80.7% attending physicians and 19.3% resident physicians. In the “after” period, there were 81.9% attendings, and 18.1% residents. Residents had higher HIV testing rates than attendings both before (RR 1.55, CI 1.46–1.66) and after (RR 1.24, CI 1.19–1.31) the implementation of the reminder. Among attendings, the HIV testing rate increased from 13.9% in the pre-reminder period to 29.5% in the post-reminder period (RR 2.14, *p* = 0.0001, CI 2.05–2.23). Among residents, the HIV testing rate increased from 21.7% in the “before” period to 36.9% after the intervention (RR 1.72, p = 0.0001, CI 1.60–1.84). The reminder resulted in a larger magnitude effect among attendings as compared to residents.

### Local considerations in HIV testing

Looking only at the time period between June 26, 2012 (the date of change in Massachusetts law requiring written consent for HIV testing), up to the date of the reminder implementation, the HIV test proportion among patients in the practice was 16.5% (4094/24842). In the study period after the reminder, this increased to 31.3% (6505/20817), *p*-value = 0.0001. These estimates are slightly higher than those we reported for the overall estimate (see Table 2) of 15.3% vs. 30.7%.

## Discussion

This study sought to evaluate the impact of a passive EMR reminder on rates of HIV testing at a hospital-based, academic primary care practice. Study results showed a two-fold increase in overall testing after reminder implementation. These results support the findings of a study from Case Western Reserve University, which showed that a passive EMR reminder decreased the number of patients never tested for HIV, as well as findings from a study at the Veterans’ Administration (VA) system, which showed increased HIV screening after implementation of a comprehensive intervention that included an active electronic reminder [10, 11]. An Indian Health Service survey also found that having an electronic reminder in place for more than one year was correlated with higher levels of HIV screening [12]. The findings are contrasted, however, by a separate, null VA study which included only low-intensity electronic reminders [13]. Overall, our results add to a growing body of literature supporting the use of EMR reminders, even for traditionally stigmatizing conditions such as HIV infection.

The reminder was particularly successful in increasing screening rates in patients with low rates of baseline testing. Older adults have been identified in the literature as a population not meeting screening goals. In the Behavioral Risk Factor Surveillance System report, Ford et al. demonstrated that HIV screening in 50–64 year olds increased slightly after the CDC recommended universal screening in 2006, but then decreased again over time to a prevalence of just 3.7% in 2010 [14]. In the current study, the reminder led to a three-fold increase in testing of patients age 46–65.

Low screening rates in older adults may reflect subconscious practitioner beliefs about the epidemiologic risk of HIV in middle-aged and older populations. However, people ≥ age 45 comprised more than one-third of new HIV diagnoses in Boston (the location of the current study) between 2009 and 2011 [15]. The discrepancy between baseline testing and local epidemiologic risk underscores the need to apply HIV screening universally. Factors such as subconscious provider or patient beliefs may influence a patient’s likelihood of being tested for HIV regardless of the EMR reminder.

We examined several other factors which may also influence the likelihood of HIV testing, including the type of provider, as well as several aspects of patient healthcare utilization. In regard to provider type, residents were more likely than attendings to test patients for HIV overall, but the reminder had less of an impact on increasing HIV testing among residents. While there could be many reasons for this, one possibility is that routine HIV screening is more a part of residents’ clinical repertoire due to the era of their training, which may lead to more comfort with the topic, regardless of an EMR reminder. All practitioners may benefit from training on who should be screened and how to discuss screening for HIV in a way that minimizes stigma, but providers with different levels of experience and training also may simply integrate new practices at different rates and use EMRs in different ways. Given that EMRs and EMR alerts have been more common since residents started training, it is also possible that they are more used to the reminders, and therefore more subject to “alert fatigue.”

Regarding healthcare utilization, frequency of patient visits was not correlated with the likelihood of receiving an HIV test, either before or after the reminder. While one may have predicted that more frequent visits would result in higher rates of HIV screening, it is possible that patients who are seen more frequently come in for more episodic visits to address acute problems, during which healthcare maintenance is not the primary goal. During such visits, due to the acute issue(s) at hand, providers may not pay attention to a passive EMR alert. In future studies, it would be useful to collect data on the reason for the visit in order to better understand this finding.

We also found that although new patients to the practice were overall more likely to receive HIV screening, the reminder had a smaller impact on increasing testing for this patient population. The finding that new patients were more likely to be screened in general may possibly be explained by the idea that addressing healthcare maintenance items is typically a routine practice for a first meeting, and providers may also feel this is a more normalized setting in which to approach a patient about HIV testing. The finding that the reminder was less effective among new patients may suggest that our estimate of the overall effect of the reminder was an overestimation of the true effect.

In general, the finding among new patients echoes a theme throughout the study, that the reminder tended to be more impactful in increasing screening rates for those patients *less* likely to get screened otherwise. This generalization is supported by the finding that patients of attendings were less likely to be screened overall as compared to patients of residents, but that the reminder led to a larger increase in HIV screening among attendings. Additionally, older patients were least likely to be screened in both time periods, yet the magnitude of the increase in screening was highest in this group. Similarly, women were less likely to be tested prior to the reminder, but the impact of the reminder was larger for women than men. While we cannot know for sure why this general trend was seen, the finding might point to the reminder serving as a normalizing factor for discussion of HIV testing with patients whom providers may not have previously thought of as “at risk,” or patients whom providers did not previously feel comfortable approaching about the topic. A potential way to improve the impact of the reminder may be to create a tiered alert in which patients who meet criteria associated with lower likelihood of screening have a higher intensity alert, such as an active reminder, which practitioners must address before being allowed to move further through the medical record.

While the robust increase in HIV screening rates after reminder implementation is very promising, it is worth noting that the practice’s overall adherence to HIV screening guidelines remained low, with 30.7% of eligible patients receiving an HIV test even after the reminder. This suggests that practices may need to consider additional strategies in order to meet screening goals. One alternative to passive EMR reminders is active reminders. The VA study discussed above, which showed a significant increase in HIV screening within its intervention arm, included an active, rather than passive, reminder [10]. Those authors also used intervention components outside the electronic reminder, including organizational factors and provider activation via educational and social initiatives. More than an electronic reminder alone may be necessary to improve screening to optimal levels.

However, a relative strength of our study is the increase in screening it achieved without any of the other time- or resource-intensive interventions described in other positive EMR reminder studies, including provider education [11], academic detailing and use of champions [10]. Indeed, our study shows a similar proportional rise in screening to at least one prior study [10] which was much more resource-intensive. In addition, most prior studies assessed screening for HIV among patients already determined to be at high risk based on pre-identified risk factors [10, 11, 13], whereas we assessed universal screening regardless of risk factors, which we think has significant implications. While the study by Avery, Del Toro, and Caron in 2014 [11] most closely matched ours in terms of the use of a passive electronic reminder and applied universal screening, the study specifically targeted clinics that were felt to be in high-risk areas, whereas our study describes an untargeted population of all risk levels.

Screening rates may also benefit from actions other than reminders that further normalize the role for HIV testing within the primary care visit. For example, Massachusetts required written consent for HIV testing until 2012, in contrast to almost any other laboratory test. Many practitioners may recall the written consent as arduous and time consuming. Actions that remove time barriers are likely to improve testing rates. Because the Massachusetts law change overlapped with our study period, this could have confounded our results. Yet, when we removed visits during the original study period prior to the change in the law, this did not reduce the effect of the intervention.

HIV screening may be further established as routine practice (and destigmatized) by maximizing use of population management support systems, such as in the medical home model, which includes screening for HIV alongside the other common screening initiatives, such as cancer screenings. In addition, “opt-out” HIV screening is also a potential way to increase screening, in which routine HIV testing is incorporated into blood draws without additional ordering required by medical providers; this has been supported by the Centers for Disease Control [16]. This method has not yet been incorporated into our practice policy. Finally, practitioners may also benefit from further education on whom to screen and how to avoid stigma in the discussion about HIV screening. Ultimately, a comprehensive approach incorporating both an EMR prompt and other behavioral strategies for practitioners may be most successful in improving screening rates.

### Limitations

The study should be viewed with several limitations. Our data collection could not capture HIV tests performed outside of our healthcare system, as practitioner behavior surrounding the manual entry of outside HIV tests and patients declining the test is variable. Therefore, our post-reminder rate of 30.7% likely underestimates the true number of patients who have met the screening goal.

We were unable to discern which practitioner-specific factors, other than level of training, may have been associated with low screening rates and exactly how the electronic reminder may have affected these specific factors. Although we could postulate that discomfort with discussing HIV screening and the impression that few of a practitioner’s patients are at risk for HIV could play a role, we cannot be certain. A final shortcoming is the relatively short period of follow up of 12 months after the implementation of the EMR reminder. Therefore, it is not clear whether the effect of the reminder will increase or decrease over time. These issues represent opportunities for further research.

## Conclusions

This study demonstrated that a passive EMR reminder doubled HIV screening rates, as well as detection of positive HIV tests, at a hospital-based primary care practice. The reminder was most effective in increasing screening among patients who were less likely to be screened due to other factors, including older age, female gender, longer enrollment in the practice, and having an attending provider. However, significant opportunity for improvement remains. Future research should examine additional patient and practitioner factors that may place patients at high risk of under-screening and pilot comprehensive interventions – in addition to EMR reminders– to improve screening rates.

## Abbreviations

CDC: Centers for Disease Control and Prevention
EMR: Electronic medical record
GEE: Generalized estimating eqs.
HIV: Human immunodeficiency virus
USPSTF: United States Preventive Services Task Force
VA: Veterans’ Administration

## References

[1] Centers for Disease Control. Monitoring selected national HIV prevention and care objectives by using HIV surveillance data—United States and 6 U.S. dependent areas—2011. In: HIV Surveillance Supplemental Reports. CDC. 2013. https://www.cdc.gov/hiv/library/reports/hiv-surveillance.html. Accessed 9 Dec 2016.

[2] Branson BM, Handsfield HH, Lampe MA, Janssen RS, Taylor AW (2006). Revised recommendations for HIV testing of adults, adolescents, and pregnant women in health-care settings. MMWR Recomm Rep.

[3] Human Immunodeficiency Virus (HIV) Infection: Screening. In: Recommendations for Primary Care Practice. United States Preventive Services Task Force. 2013. http://www.uspreventiveservicestaskforce.org/uspstf/uspshivi.htm. Accessed 21 Oct 2015.

[4] Libman H (2011). Clinical crossroads. Screening for HIV infection: a healthy, “low-risk” 42-year-old man. JAMA.

[5] Chaudhry R, Scheitel SM, McMurtry EK, Leutink DJ, Cabanela RL, Naessens JM (2007). Web-based proactive system to improve breast cancer screening: a randomized controlled trial. Arch Intern Med.

[6] DeJesus RS, Angstman KB, Kesman R, Stroebel RJ, Bernard ME, Scheitel SM (2012). Use of a clinical decision support system to increase osteoporosis screening. J Eval Clin Pract.

[7] Chaudhry R, Tulledge-Scheitel SM, Parks DA, Angstman KB, Decker LK, Stroebel RJ (2012). Use of a web-based clinical decision support system to improve abdominal aortic aneurysm screening in a primary care practice. J Eval Clin Pract.

[8] Schriefer SP, Landis SE, Turbow DJ, Patch SC (2009). Effect of a computerized body mass index prompt on diagnosis and treatment of adult obesity. Fam Med.

[9] Sidlow R, Msaouel P. Improving hepatitis C virus screening rates in primary care: a targeted intervention using the electronic health record. J Healthc Qual. 2015;37(5):319–23. 10.1097/JHQ.0000000000000010 26186704

[10] Goetz MB, Hoang T, Bowman C, Knapp H, Rossman B, Smith R (2008). A system-wide intervention to improve HIV testing in the veterans health administration. J Gen Intern Med.

[11] Avery AK, Del Toro M, Caron A (2014). Increases in HIV screening in primary care clinics through an electronic reminder: an interrupted time series. BMJ Qual Saf.

[12] Reilley B, Leston J, Tulloch S, Neel L, Galope M, Taylor M. Implementation of national HIV Screening recommendations in the Indian Health Service. J Int Assoc Provid AIDS Care. 2015;14(4):291–4. 10.1177/2325957415570744 PMC6749603 25656861

[13] Sundaram V, Lazzeroni LC, Douglass LR, Sanders GD, Tempio P, Owens DK (2009). A randomized trial of computer-based reminders and audit and feedback to improve HIV screening in a primary care setting. Int J STD AIDS.

[14] Ford CL, Mulatu MS, Godette DC, Gaines TL (2015). Trends in HIV testing among U.S. older adults prior to and since release of CDC’s routine HIV testing recommendations: national findings from the BRFSS. Public Health Rep.

[15] Massachusetts Department of Public Health (MDPH). Regional HIV/AIDS Epidemiologic Profile of City of Boston, Massachusetts: 2013. MDPH 2013. http://www.mass.gov/eohhs/docs/dph/aids/2013-profiles/city-boston.pdf. Accessed 9 Dec 2016.

[16] Branson BM, Handsfield HH, Lampe MA, Janssen RS, Taylor AW, Lyss SB, Clark JE. Revised Recommendations for HIV Testing of Adults, Adolescents, and Pregnant Women in Health-Care Settings. Centers for Disease Control. 2006. https://www.cdc.gov/mmwr/preview/mmwrhtml/rr5514a1.htm. Accessed 29 Sept 2017. 16988643

